# Three-dimensional quantitative analysis of adhesive remnants and enamel loss resulting from debonding orthodontic molar tubes

**DOI:** 10.1186/1746-160X-10-37

**Published:** 2014-09-10

**Authors:** Joanna Janiszewska-Olszowska, Katarzyna Tandecka, Tomasz Szatkiewicz, Katarzyna Sporniak-Tutak, Katarzyna Grocholewicz

**Affiliations:** 1 Department of General Dentistry Pomeranian Medical, University of Szczecin, al. Powstancow Wlkp. 72, 70-111 Szczecin, Poland; 2 Department of Fine Mechanics Koszalin, University of Technology, ul. Raclawicka 15-17, 75-620 Koszalin, Poland; 3 Department of Dental Surgery Pomeranian Medical, University of Szczecin, al. Powstancow Wlkp. 72, 70-111 Szczecin, Poland

**Keywords:** Orthodontic debonding, Enamel damage, Resin remnants

## Abstract

**Aims:**

Presenting a new method for direct, quantitative analysis of enamel surface. Measurement of adhesive remnants and enamel loss resulting from debonding molar tubes.

**Material and methods:**

Buccal surfaces of fifteen extracted human molars were directly scanned with an optic blue-light 3D scanner to the nearest 2 μm. After 20 s etching molar tubes were bonded and after 24 h storing in 0.9% saline - debonded. Then 3D scanning was repeated. Superimposition and comparison were proceeded and shape alterations of the entire objects were analyzed using specialized computer software. Residual adhesive heights as well as enamel loss depths have been obtained for the entire buccal surfaces. Residual adhesive volume and enamel loss volume have been calculated for every tooth.

**Results:**

The maximum height of adhesive remaining on enamel surface was 0.76 mm and the volume on particular teeth ranged from 0.047 mm^3^ to 4.16 mm^3^. The median adhesive remnant volume was 0.988 mm^3^. Mean depths of enamel loss for particular teeth ranged from 0.0076 mm to 0.0416 mm. Highest maximum depth of enamel loss was 0.207 mm. Median volume of enamel loss was 0.104 mm^3^ and maximum volume was 1.484 mm^3^.

**Conclusions:**

Blue-light 3D scanning is able to provide direct precise scans of the enamel surface, which can be superimposed in order to calculate shape alterations. Debonding molar tubes leaves a certain amount of adhesive remnants on the enamel, however the interface fracture pattern varies for particular teeth and areas of enamel loss are present as well.

## Introduction

Bonded molar tubes cover a significant proportion of their buccal surface. After active treatment termination they are debonded, usually using ligature-cutting pliers.

The mode of bond failure has been classified by Strattman et al. [1] into four types:

1. fracture interface lying between adhesive and bracket base

2. fracture interface lying between adhesive and enamel surface

3. fracture interface lying partly between adhesive and enamel surface and partly within the adhesive

4. fracture interface lying partly between adhesive and enamel surface, partly within the adhesive and partly between adhesive and bracket base

One of the factors determining different modes of adhesive failure during debonding may be enamel morphology varying between tooth groups. Adhesive remnant index (ARI) according to Årtun and Bergland [2] is assessed according to the following criteria 0 – no adhesive left, 1 – less than half of the adhesive left, 2 – more than half of the adhesive remained, 3 – all adhesive left. According to Krell et al. [3] adhesive remnant index criteria are as follows: 1. All adhesive remains on the tooth, 2. More than 90% adhesive remains on the tooth, 3. More than 10%, but less than 90% adhesive remains on the tooth, 4. Less than 10% adhesive remains on the tooth, 5. No adhesive remains on the tooth. Osorio et al. [4] have calculated Adhesive Remnant Index (ARI) as the quotient of area of residual resin on the tooth and the area of bracket base in percentage.

Although used by numerous authors [4-15] ARI is only a surface – assessment measurement and does not measure the volume of resin left on the teeth. Another method of quantitative residual adhesive assessment has been used by David et al. [16], who have calculated resin remnant weight.

The process of debonding results not only in adhesive remnants, but also causes irreversible enamel loss [1,11,15,17]. The failure of the interface between adhesive and enamel removes a layer of enamel.

The aim of the study was to quantitatively assess the amount of enamel loss and adhesive remnants following the debonding of molar tubes.

## Material and methods

This study has been decided to be exempt from approval by the bioethical committee of our university (decision reference No: KB-0012/09/01/2013).

Experimental teeth were human third molars extracted for orthodontic reasons from patients aged 16–24 years. All of them have been inspected for soundness of buccal surface and fifteen teeth free of carious lesions, cracks or restorations have been selected. They were stored in distilled water for 24 hours before bonding. After cleaning with a low speed bristle brush and non-fluoride pumice slurry, rinsing for 10 seconds and air-drying with oil-free compressed air, they have been numbered in sequence and for the purpose of 3D scanning embedded in impression silicone (Bisico S1 Soft, Bisico, Germany) in order to prevent unnecessary movement during manipulation.

In order to check if the sample size is appropriate, an on-line power and sample size calculator was used [18]. The threshold value of clinical significance has been set at 0.05 mm both for adhesive remnants and enamel loss. At the level of significance alfa = 0.05 and at the power of the test of 0.80, the sample size yielded 13 and 14, respectively.

**Figure 1:**
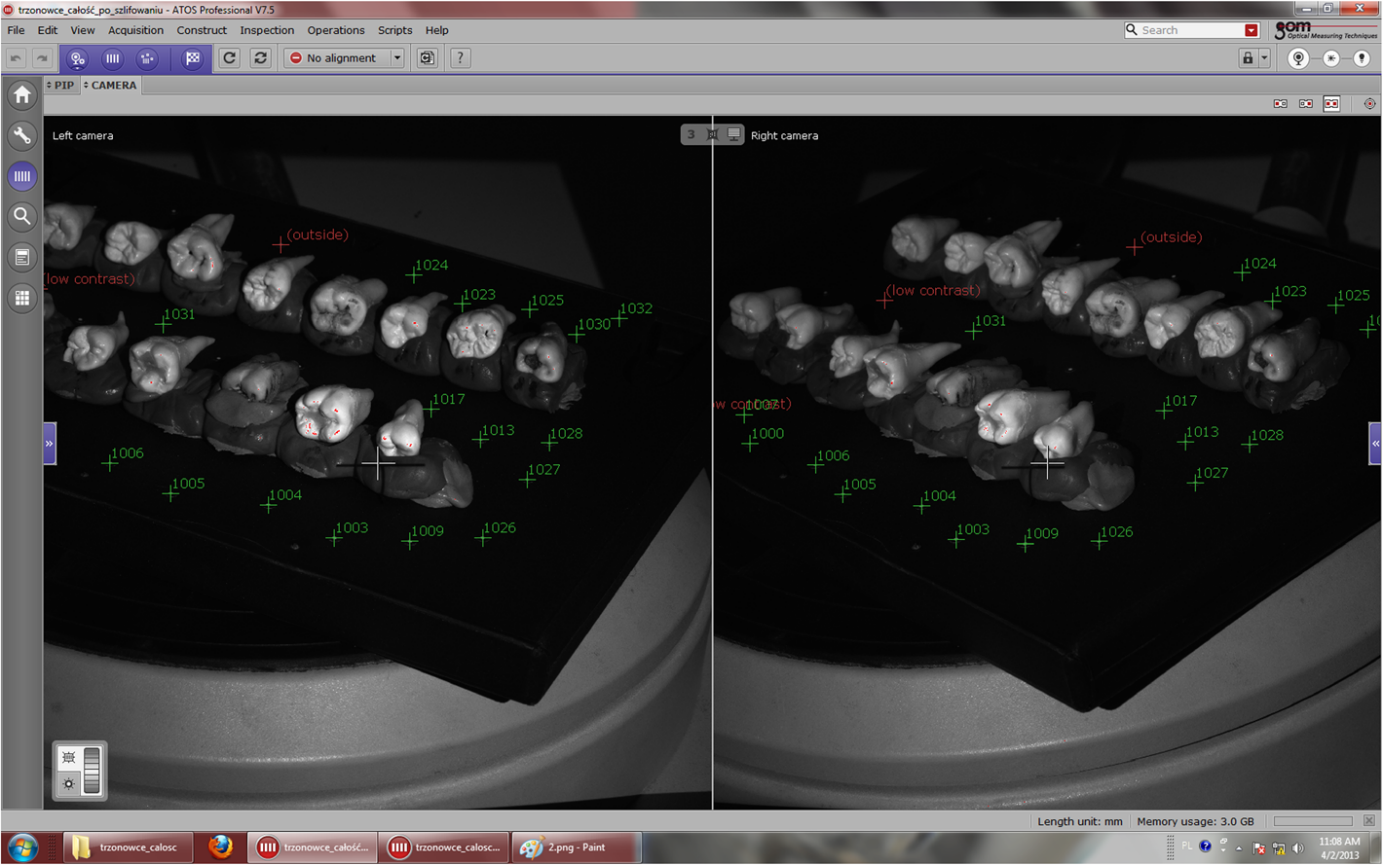
The process of blue-light 3D scanning.

**Figure 2:**
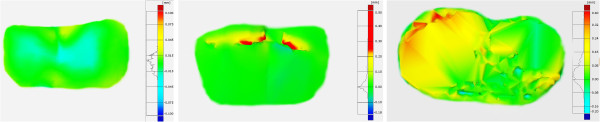
Shape alteration analysis in GOM Inspect software (teeth Ref. No. 1–3).

The area to be bonded was determined as centre of the buccal surface, parallel to the crown long axis in order to simulate the clinical conditions. After 20 seconds etching with 35% phosphoric acid (Ultra Etch, Ultradent, USA), 10 seconds rinsing with water spray and drying with oil-free compressed air, molar tubes (ERA, Farfield, USA) were bonded directly using chemical-cure orthodontic adhesive (Unite, 3 M, USA). They were firmly pressed onto the enamel in order to minimize excess material. Excess resin on the margins of the molar tubes was carefully removed with a microbrush. After 10 minutes setting, the teeth with molar tubes bonded were stored in 0.9% saline solution for 24 hours, rinsed with distilled water to prevent saline crystallization and debonded using ligature cutting pliers positioned occlusally and gingivally in order to gently peel the molar tubes from enamel, similarly as in the clinical conditions.In order to quantitatively analyze resin remnants and enamel loss, buccal surfaces of all teeth were scanned with a 3D optical scanner (Atos III, Triple Scan, GOM, Germany) before etching, and after debonding, using a lens of the field of 170×130×130 mm. Atos scanner uses the technique of triangulation: two cameras observe the course of stripes projected on the object measured and for each pixel of camera sensor point’s coordinations are calculated with high precision (Figure 1). The resulting virtual elements were transformed into reference elements. The precision of the scanner (2 μm) is maintained by regular calibration procedure thus error study was not performed.Alteration of the macrogeometric features of enamel surface resulting from orthodontic debonding has been analyzed using GOM Inspect software (GOM, Braunschweig, Germany) allowing to inspect digitalized models measured by the use of Atos Triple Scan with reference models. Scans of pretreatment enamel surfaces were used as reference for comparisons. Every point of the nominal data has been compared with reference data, thus calculating shape alteration of the entire object analyzed (Figure 2). This analysis enabled calculating residual adhesive volume and heights in different locations of the buccal surface. Superimposition and comparison were proceeded using teeth before bonding as reference and teeth with molar tubes removed – as virtual objects. GOM Inspect software allowed to calculate both adhesive remnants and enamel loss.

## Results

Visual macroscopic assessment of adhesive failure on the molars following molar tubes debonding revealed no adhesive remnants on three of the specimens assessed (Ref. No. 1, 10 and 15). Complete coverage of the bonding area with adhesive was found on six teeth (Ref. No. 3, 5, 6, 7, 8, 9). Six teeth had some remnants, whereas some adhesive was visible on the molar tubes bases.

**Figure 3:**
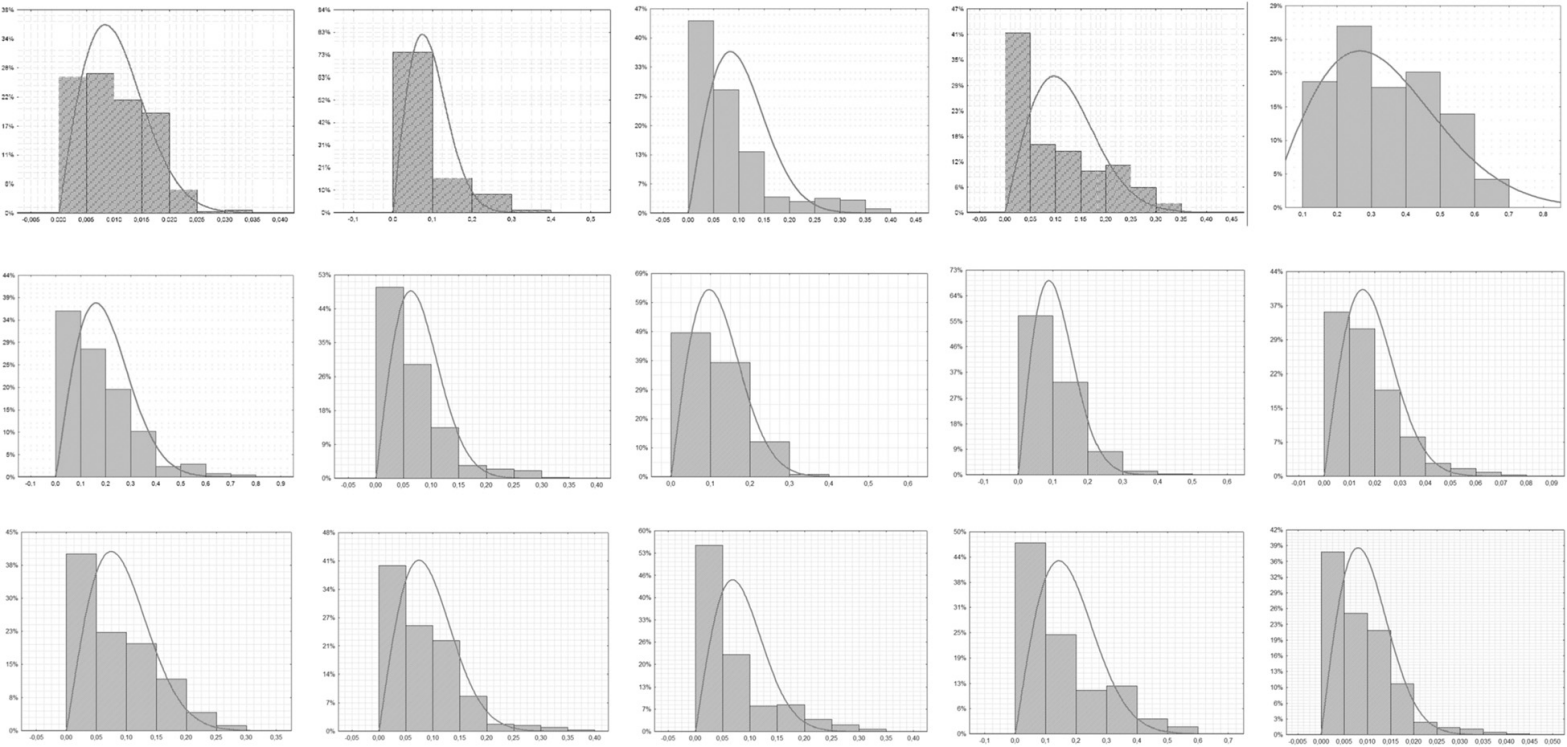
Histograms of adhesive remnants on particular teeth; x-height, y – percentage of observations; first row: teeth No 1–5, second row: teeth 6–10, third row: teeth 11–15.

The results concerning the amount of adhesive after debonding have been presented in Table 1. The volume of adhesive remaining on particular teeth ranged from 0.047 mm^3^ to 4.16 mm^3^. The median of adhesive remnant volume was 0.988 mm^3^. The distribution of adhesive remnants on particular teeth has been presented in Figure 3.

**Table 1:** Adhesive remnants on particular teeth after debonding

**Tooth number**	**Adhesive remnants**				
	**Height [mm]**				**Volume [mm**^**3**^**]**
	**Mean**	**SD**	**Max**	**Min**	
1	0.0099	0.0063	0.0325	1.5E-5	0.047
2	0.0688	0.0779	0.4251	0.0001	0.295
3	0.0952	0.0776	0.4061	2.7E-5	1.12
4	0.1349	0.0997	0.372	3.7E-5	0.678
5	0.238	0.1668	0.6986	0.0004	3.24
6	0.1492	0.1361	0.7606	4E-6	4.16
7	0.0662	0.0585	0.3323	0.0001	0.35
8	0.084	0.0584	0.3764	2.5E-5	1.52
9	0.1	0.0721	0.4727	0.0001	1.234
10	0.0168	0.013	0.0766	5.7E-5	0.271
11	0.0861	0.0646	0.2958	4.2E-5	1.256
12	0.0815	0.0649	0.3609	5.8E-5	0.988
13	0.065	0.0698	0.3296	9.5E-5	0.148
14	0.1518	0.1305	0.5752	0.0002	1.224
15	0.0087	0.007	0.0411	1.6E-5	0.108

**Figure 4:**
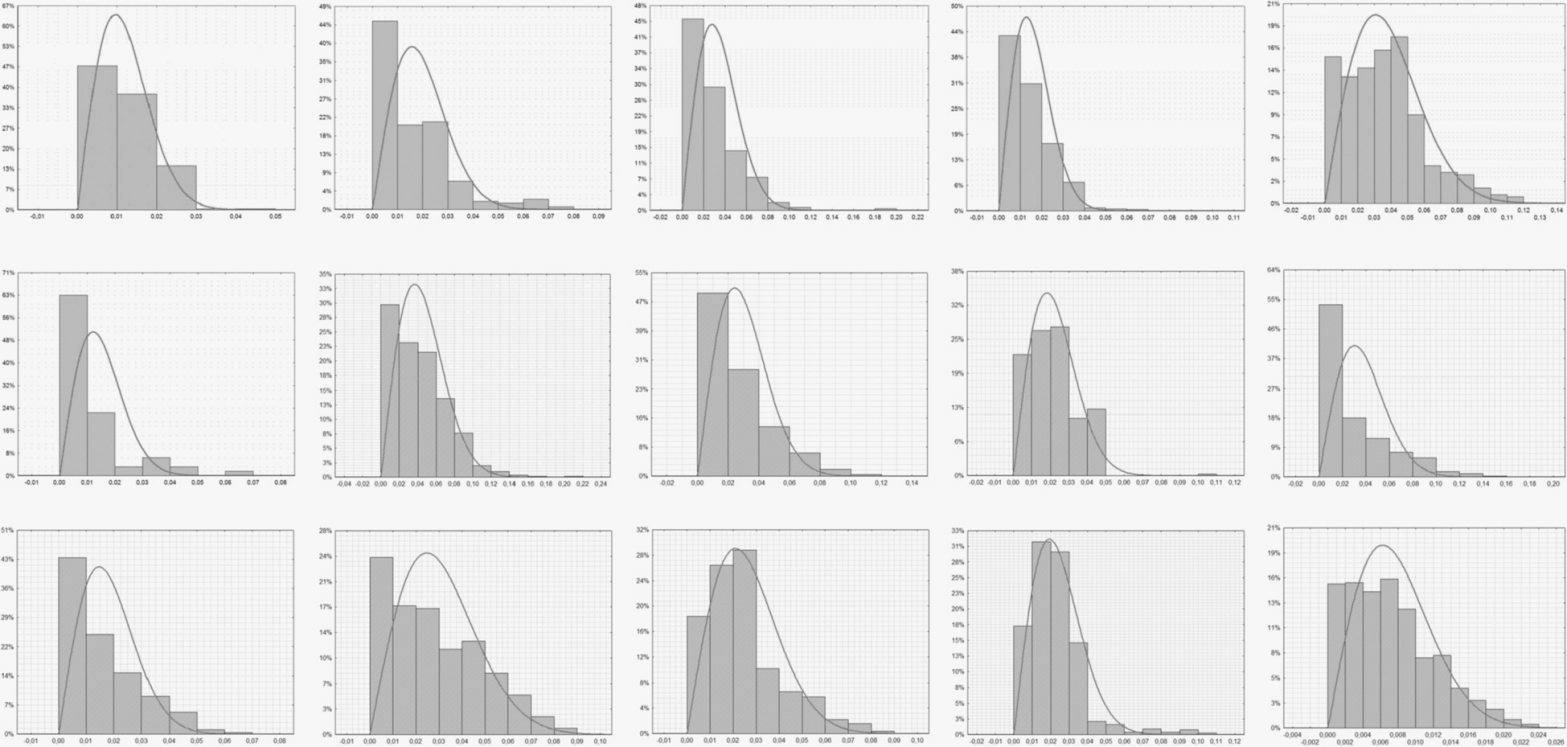
Histograms of enamel loss on particular teeth; x-depth, y – percentage of observations; first row: teeth No 1–5, second row: teeth 6–10, third row: teeth 11–15.

Enamel loss depth and volume for particular teeth have been presented in Table 2. Maximum depth was 0.207 mm. Mean volume for all the teeth was 0.104 mm^3^ and maximum volume 1.484 mm^3^. Extremely low values of adhesive remnant height or enamel loss depth are within the measurement error. The distribution of enamel loss on particular teeth has been presented in Figure 4.

**Table 2:** Enamel loss on particular teeth after debonding

**Tooth number**	**Enamel loss**				
	**Depth [mm]**				**Volume [mm**^**3**^**]**
	**Mean**	**SD**	**Max**	**Min**	
1	0.0113	0.0074	0.0435	0.0001	0.076
2	0.0163	0.0148	0.073	1.4E-5	0.327
3	0.0326	0.0219	0.1394	2.4E-5	0.104
4	0.0142	0.0127	0.0975	4E-6	0.419
5	0.0387	0.0298	0.155	5.4E-5	0.033
6	0.0134	0.0123	0.0654	6.7E-5	0.002
7	0.0416	0.03	0.2071	0.0002	0.45
8	0.0263	0.0226	0.1266	3.7E-5	0.065
9	0.0213	0.0138	0.1027	0.0002	0.037
10	0.0305	0.0302	0.1677	1E-6	1.484
11	0.0149	0.0125	0.0662	2.4E-5	0.161
12	0.0282	0.0203	0.0857	8E-8	0.238
13	0.0246	0.0165	0.0843	0.0002	0.087
14	0.0223	0.0153	0.1083	4.4E-5	0.098
15	0.0076	0.0069	0.0375	1.4E-5	0.15

## Discussion

Enamel damage may result from enamel cracking during debonding procedure or grinding residual adhesive after debonding. The area of enamel damage and the volume of enamel loss are dependent on the bracket square surface size [11]. It could be thus less detrimental to use smaller brackets, however bond strength might then be compromised. The molar tubes used in the present study have a wide base and in our opinion, a good fit to the buccal surface, allowing for good bond strength, however the area subjected to treatment alteration covers a substantial proportion of the entire buccal surface.

The 3D blue-light scanning allowed to calculate the height and volume of adhesive remnants avoiding sample sputtering, thus allowing to analyze the enamel surface before etching, and after debonding. Two studies [19,20] quantitatively assessing adhesive remnants and enamel damage on molars has been found, both proceeded using a 3D laser scanning. Direct analysis of enamel surface was impossible due to light reflection [21]. Thus plaster models of the surfaces were made and scanned, causing an inevitable measurement error. This disadvantage was reduced in the present study by using blue light technology, producing a high measurement accuracy of shiny surfaces. Experiment conditions different from an in vivo situation constitute a disadvantage of direct assessing enamel surface mainly due to humidity, which influences bond strength. It has been supported, that in vitro bond strength is higher than that measured in vivo [22].

Debonding with ligature cutting pliers, although simulates the clinical procedure, does not guarantee standarization [12]. This fact was confirmed in the present study, a diversity of debonding patterns was seen between individual teeth.

In the present study mean height of composite remnants on particular teeth ranged from 0.0087 mm to 0.238 mm; mean value: 0.084 mm, which is less than mean 200.2 μm reported by Ryf et al. [20]. In the present study mean volume of residual composite was 1.1 mm^3^. A higher value (2.48 mm^3^) has been reported by Ryf et al. [20].

In the present study a mean enamel loss for particular teeth ranged from 0.0076 mm to 0.0416 mm, which is less than the mean depth of 44.9 μm reported by Ryf et al. [20]. The deepest enamel loss of 297.8 μm [20] is higher than 0.2071 mm from the present study. Median enamel loss volume in the present study was 0.104 mm^3^, which is more than 0.02 mm^3^ reported by Ryf et al. [20]. The factors causing discordance between the results could be different materials used (brackets and adhesive), different experiment conditions and different methods of measurements or calculation.

Acid etching and bracket bonding result in resin infiltration into the enamel [23]. It could be supposed that in order to completely remove adhesive remnants, a superficial enamel layer should be removed.

In the present study - debonding with adhesive-enamel bond failure results in adhesive microremnants on the enamel. This is supporting earlier findings [24-26] that macroscopically clean enamel surface is covered by a thin layer of adhesive and underlying the necessity of complete adhesive removal to avoid plaque accumulation and discoloration. Unfortunately, surface scanning does not allow to assess the depth or volume of resin remnants within the enamel (resulting from etching and infiltration).

Al Shamsi et al. [19] have reported a mean remnant adhesive thickness of 31.2 μm with light cure adhesive and 102.7 μm with adhesive precoated brackets. Enamel loss directly after debonding was not reported. However, according to Alessandri Bonetti et al., [8] bracket precoating had no effect on adhesive remnant index after debonding. However, Kinch et al. [7] found that ARI is dependent on bracket type.

In theory, the bond failure may occur between the bracket base and composite, between the composite and enamel or within the composite (cohesive bond failure). The brackets are removed using pliers exerting a combination of shear, tensile and torque forces [11]. Bond failure between the adhesive and enamel may result in a certain amount of enamel substance loss [16]. Pont et al. [11] using energy dispersive x-ray spectrometry mean area scanning analysis have supported elemental loss of calcium from enamel.

However, in most cases some adhesive is present on the tooth after bracket debonding, which is mechanically removed. The accuracy of the Atos scanner declared by the manufacturer and maintained by regular calibration is 2 μm. A similar level of precision has been reported by van Vaes et al. [27]. However, no resin remnants or enamel loss directly after debonding were calculated. Adhesive height and volume has an influence on the wear of the tool used for its removal, whereas surface area influences the volume of potential enamel loss.

The results of the present study indicate that the bond failure occurring visually at the interface between bonding material and enamel results in a certain amount of enamel loss. The debonding pattern is thus a cohesive failure of the adhesive within the enamel, therefore both enamel loss and resin remnants may be seen on the same surface fragment. In order to completely remove remnant resin, clean-up procedure should be performed even on macroscopically adhesive-free surfaces. An alternative could constitute leaving some composite remnants, but highly polished.

## Conclusions

1. Blue-light 3D scanning is able to provide direct precise scans of the enamel surface, which can be superimposed in order to calculate shape alterations. This method can be recommended for future research.

2. Debonding molar tubes leaves a certain amount of adhesive remnants on the enamel, however the interface fracture pattern varies for particular teeth and areas of enamel loss are present as well.

## Competing interests

The authors declare that they have no competing interests.

## Authors’ contributions

JJO study conception and design, data interpretation, writing manuscript. KT 3D scanning and data processing, drawing figures, participation in data interpretation. TS 3D scanning, participation in data interpretation. KST providing material for the study (extracted teeth), participation in manuscript preparation. KG critical revising for intellectual content. All authors have read and approved the final version of the manuscript.

## Authors’ information

JJO is a practising orthodontist and assistant professor Department of General Dentistry. KT and TS are engineer scientists and assistant professors Department of Fine Mechanics. KST is a practising specialist in both oral and maxillofacial surgery, associate professor Department of Dental Surgery. KG is a practising specialist in prosthetic dentistry, head of an interdisciplinary Department of General Dentistry.
